# Spatial proteomic and phospho-proteomic organization in three prototypical cell migration modes

**DOI:** 10.1186/1477-5956-12-23

**Published:** 2014-05-01

**Authors:** Georgios Fengos, Alexander Schmidt, Katrin Martin, Erika Fluri, Ruedi Aebersold, Dagmar Iber, Olivier Pertz

**Affiliations:** 1 ETH Zurich, D-BSSE, Mattenstrasse 26, CH-4058 Basel, Switzerland; 2 University of Basel, Biozentrum, Klingelbergstrasse 50/70, CH-4056 Basel, Switzerland; 3 Department Biomedicine, University of Basel, Mattenstrasse 28, CH-4058 Basel, Switzerland; 4 ETH Zurich, Department of Biology, Institute of Molecular Systems Biology, Wolfgang-Pauli-Strasse 16, CH-8093 Zurich, Switzerland; 5 University of Zurich, Faculty of Science, Zurich, Switzerland

**Keywords:** Fibroblast, Directional cell migration, Signaling, Proteomics, Phosphorylation

## Abstract

**Background:**

Tight spatio-temporal signaling of cytoskeletal and adhesion dynamics is required for localized membrane protrusion that drives directed cell migration. Different ensembles of proteins are therefore likely to get recruited and phosphorylated in membrane protrusions in response to specific cues.

**Results:**

Here, we use an assay that allows to biochemically purify extending protrusions of cells migrating in response to three prototypical receptors: integrins, recepor tyrosine kinases and G-coupled protein receptors. Using quantitative proteomics and phospho-proteomics approaches, we provide evidence for the existence of cue-specific, spatially distinct protein networks in the different cell migration modes.

**Conclusions:**

The integrated analysis of the large-scale experimental data with protein information from databases allows us to understand some emergent properties of spatial regulation of signaling during cell migration. This provides the cell migration community with a large-scale view of the distribution of proteins and phospho-proteins regulating directed cell migration.

## Background

Recent advances in biomedical research have increased the throughput of data generation for the characterization of biological systems and their functions in terms of the proteins that are involved. Although the whole proteome of an organism reflects the full set of proteins encoded in a given genome, not all of these proteins coexist at all times and at equal levels. Cell protein abundance varies according to different physiological or pathological conditions [1,2]. Thus the protein levels at a given instance reflect the dynamics of gene regulation. Identification and quantification of protein levels under different conditions should therefore help constructing a comprehensive database of protein sets leading to possible biological outcomes/functions. Additional information of the post-translational modifications of these proteins in terms of phosphorylation is required to provide a specific signaling context to the role of identified proteins [3]. Network information about protein signaling interactions combined with the experimentally measured data enables the identification of protein ensembles that regulate a specific function. In this study, the function that is under focus is cell migration as a response to different molecular stimuli.

**Figure 1:**
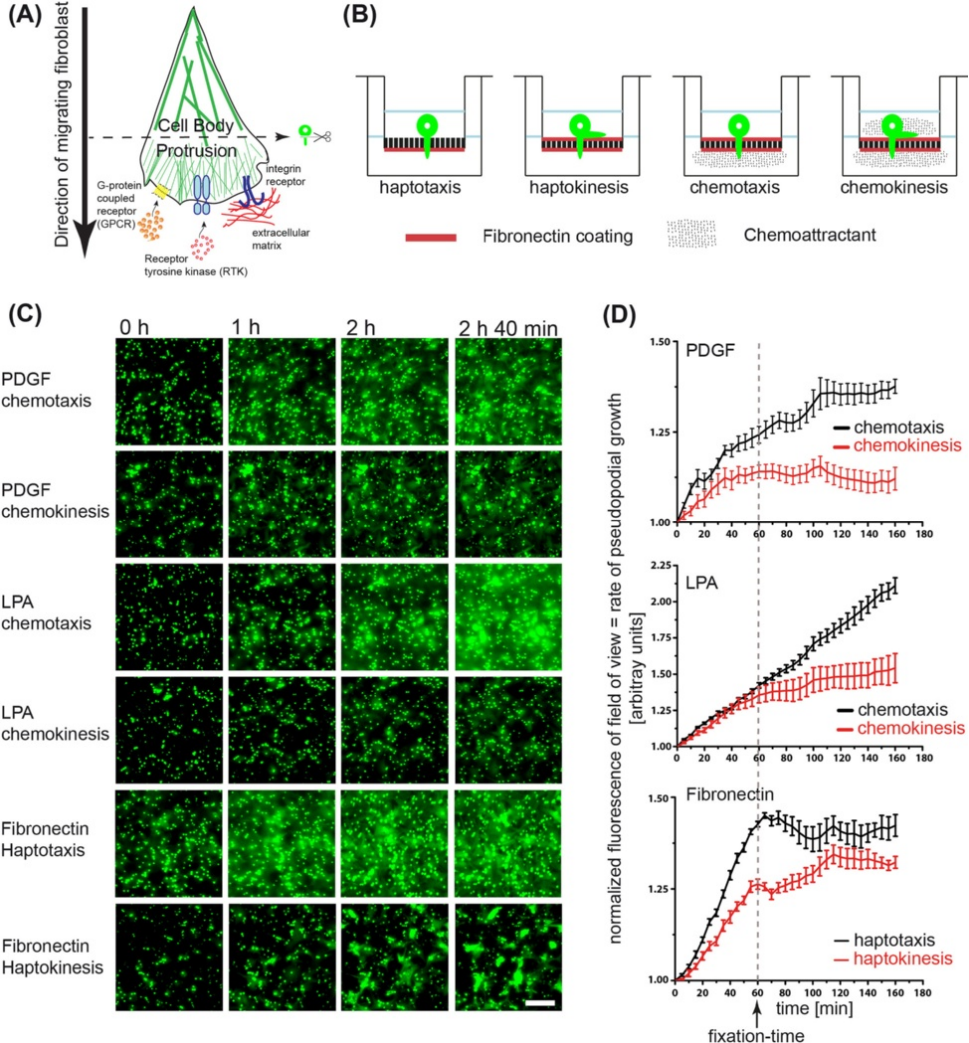
**Pseudopod purification system. (A)** Cartoon of directional migration in response to different cues. Integrins, RTKs or GPCR can all induce directional cell migration. **(B)**. Different experimental strategies to induce hapto- or chemo- taxis directional, versus control hapto- or chemo- kinesis random cell migration on 3 μm transwell filters. **(C,D)**. Pseudopod extension dynamics on bottom filter surface. **(C)** Live imaging of pseudopod protrusion of bottom transwell surface. Serum-starved, Celltracker-labeled fibroblasts, were allowed to attach on the filter for one hour, stimulated with or not with chemoattractants and timelapse imaging was started for 3 hours. Bar = 50 μm. **(D)**. Quantification of pseudopodial extension. Fluorescent intensity on the bottom filter was integrated and normalized to time point 0. Normalized signal indicates kinetics of membrane protrusion.

Directed migration is a result of the ability of cells to sense shallow differences in the concentration of a chemical cue and translate them into a steep signaling response which orients the polarity of the cell [4,5]. Morphologically, cells respond by polymerizing actin to deform the membrane, enabling to extend local protrusions, which are stabilized through adhesive contacts with the extracellular matrix towards the attractive stimulus [6,7]. This membrane protrusion process also depends on microtubule dynamics [8], as well as membrane trafficking [9], allowing regulation of membrane remodelling. Because directed membrane protrusion necessitates exquisite crosstalk between actin, adhesion, microtubule and trafficking dynamics, it is reasonable to assume that this process involves precise spatio-temporal regulation of whole signaling networks. This hypothesis prompted us to compare the pool of proteins of the protrusion with the pool of proteins from the cell-body (Figure 1A). By coupling assays, that allow the biochemical purification of pseudopods and cell-body of migrating cells, with quantitative proteomics approaches, a highly polarized distribution of specific proteins were observed in pseudopod and cell-body fractions [10]. Similar results have also been observed upon proteomic analysis of purified neurite and soma fractions of differentiating neuronal-like cells [11]. By providing comprehensive lists of proteins that localize to specific subcellular compartments, these approaches allow for an integrated view of spatio-temporal signaling networks that underlie cell migration and neuronal differentiation.

While we know a lot about the signaling events that regulate cytoskeletal and adhesion dynamics during cell migration, one current problem is that this knowledge results from a large variety of different experimental systems, making it difficult to pinpoint context-dependent signaling networks inherent to different cell migration modalities. Cells can migrate in response to different extracellular stimuli, whose asymmetric distribution provides directionality. Migration in response to gradients of immobilized extracellular matrix (ECM) components, also called haptotaxis, is thought to involve mechanosensing through adhesion molecules called integrins [12]. Migration in response to gradients of soluble chemokines, also called chemotaxis, involves growth factor receptors, which will, in turn, modulate integrin adhesion dynamics to orient the directed cell migration process. Different receptor systems, such as receptor tyrosine kinase (RTK) and G-coupled protein receptors (GPCR), are able to sense distinct chemokines to induce polarized cell migration. Due to the distinct signaling programs that both receptors are known to activate, different signaling networks are likely to be recruited to the leading edge in response to distinct cues that activate each receptor system. From an experimental perspective, it is noteworthy to mention that when these cues are applied homogeneously to cells, random migration occurs. Random cell migration occurring on homogeneous ECM is then termed haptokinesis, while bulk growth factor stimulation leads to chemokinesis.

Here we compare proteomes and phospho-proteomes of pseudopod and cell-body of NIH 3 T3 fibroblasts that directionally migrate in response to three prototypical extracellular cues involving three different receptor systems: an integrin, an RTK or a GPCR. For that purpose, we engineered pseudopod purification assays for NIH 3 T3 fibroblasts that directionally extend pseudopods in response to a fibronectin gradient (haptotaxis), a platelet derived growth factor (PDGF) gradient (PDGF chemotaxis) or a Lysophosphatidic acid (LPA) gradient (LPA chemotaxis). We then used quantitative proteomics techniques to identify pseudopod and cell-body proteomes and phospho-proteomes in the three cell migration modes. We find that specific proteins are recruited to and phosphorylated in the pseudopod depending on the cue that drives the prototypical modes of directional cell migration. This provides a resource for the cell migration community to explore cue-specific spatio-temporal signaling networks within one specific cell system.

## Results

To purify pseudopods and cell-body in the three cell migration modes (Figure 1A), we adapted a previously described assay that consists of a microporous transwell filter to purify pseudopods from the cell-body [10]. In this assay, the 3 µm pores of the transwell filter allow protrusion of pseudopods but hinder the translocation of the cell-body. Because the pseudopod only represents a small amount of the total cell mass, it is not possible to directly compare absolute protein abundance in both fractions on a per cell basis. To estimate relative protein enrichment in the pseudopod and cell-body fractions, we compared equal protein amounts of both fractions, as in previous studies [10,11]. A simple ratio of protein abundance in both fractions therefore represents the relative density of a protein in pseudopods or cell-body.

The modified original assay used for the induction directional pseudopod protrusion in response to different extracellular cues, is shown in Figure 1B [10]. For the haptotaxis cell migration model, the bottom surface of the filter was coated with fibronectin, and for the chemotaxis cell migration system, the top and bottom filter surface were coated with fibronectin. Directionality was imposed by adding chemokines (PDGF or LPA) to the bottom (chemotaxis) or the top and bottom compartments (chemokinesis). Chemokines were absent in the hapto -taxis or -kinesis experimental systems. Fluorescence time-lapse monitoring the membrane protrusion was started one hour after the attachment time, when chemokines were added (Figure 1C). An additional movie file shows this in more detail (Additional file 1). Fluorescence intensity per field of view was integrated over time and represents the rate of membrane protrusion on the bottom filter surface (Figure 1D).

During haptokinesis, uncoordinated protrusion/retraction events led to inefficient global membrane protrusion to the bottom filter surface. Similar results were observed for the PDGF and LPA chemokinesis controls. During haptotaxis, synchronized membrane protrusion without retraction events allowed efficient global membrane protrusion to the bottom filter surface. We observed that the rate of membrane protrusion followed a linear progression up to two hours after plating. PDGF chemotaxis immediately led to very efficient membrane protrusion that subsequently dampened, but were still strong 1 hour after stimulation, especially when compared to the chemokinesis control. LPA chemotaxis led to a continuous linear increase of the fluorescent protruding membrane, which was not observed during the chemokinesis control. These experiments show that robust and synchronized membrane protrusion can be observed in each of the cell migration modes up to one hour after growth factor stimulation in the chemotaxis, and up to 2 hours in the haptotaxis experimental systems. Thus, this allows purification of these pseudopods in a defined morphodynamic state of protrusion.

**Figure 2:**
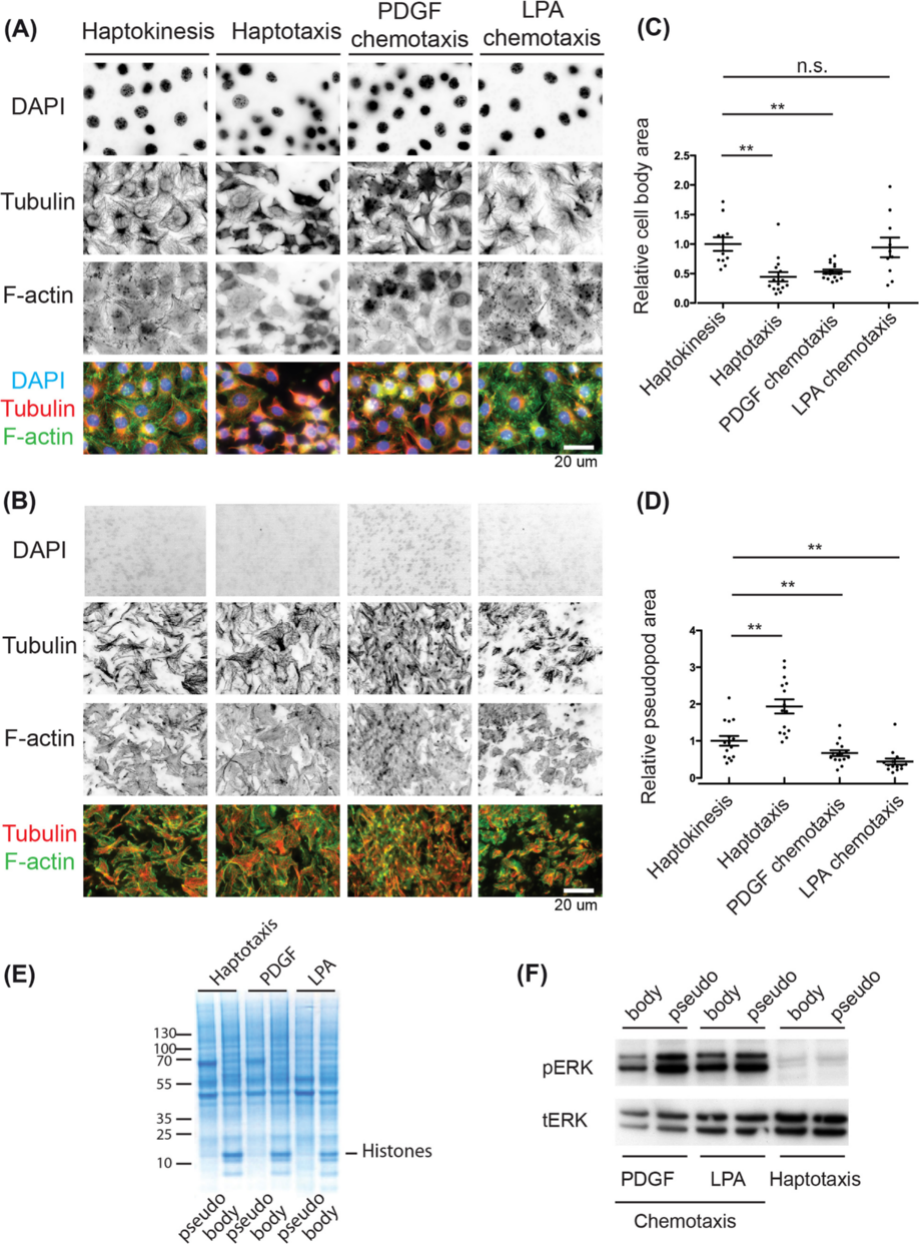
**Further characterization of pseudopod purification system. (A,B)** Cell body and pseudopod morphologies in the different cell migration systems. Cells were allowed to attach for 1 hour, then treated with or not with chemoattractants for another hour. Filters were then fixed and stained. Pseudopods (bottom filter) or cell bodies (top filter) were then scratched away; filters were mounted and evaluated using epifluorescence microscopy with a 60x high numerical aperture. Representative micrographs indicating nuclei in blue (DAPI), tubulin in red (α-tubulin), and F-actin in green (phalloidin) are shown. Single channels in inverted black and white contrast, as well as color composites are shown. Bar = 20 µm. **(A)** Cell body morphology. **(B)** Pseudopod morphology. **(C,D)** Quantification of relative cell body (top filter surface) or pseudopod (bottom filter surface) spreading. Region of interest in the images shown in **(A,B)** were manually drawn, and area was measured. Area was then normalized to the average cell body or pseudopod areas observed in haptokinesis. Average as well as standard deviations are shown. Statistical significance is indicated (T-test, **: p < 0.05, n.s.: non-significant). **(C)** Cell body area. **(D)** Pseudopod area. **(E,F)** Biochemical analysis of pseudopod and cell body fractions in the different cell migration modes. Equal amounts of cell body and pseudopod fractions were analyzed. **(E)** Coomassie stained gel of pseudopod and cell body fractions in the different cell migration modes. **(F)** ERK activation status in the different pseudopod and cell body fractions. Different fractions were probed with anti phospho-ERK and total-ERK antibodies. Note that phospho-ERK and total-ERK signals comes from two independent western blots. Raw data is shown in supplementary Figure S5.

We then performed a series of high-resolution immunostaining experiments in which we labeled the F-actin (green) and microtubule cytoskeletons (red), as well as the nucleus (blue) (Figure 2A-B). We only observed nuclei on the top filter surface, indicating that cell bodies cannot squeeze to the bottom surface (compare Figure 2A and B). In haptokinesis, fibroblasts were able to spread well on the fibronectin on the top surface. Consistently with the absence of fibronectin on the top surface, lower levels of spreading and F-actin were observed in haptotaxis. In the chemotaxis experimental systems, LPA led to a slight increase in spreading while PDGF decreased the amount of spreading and F-actin content on the top filter surface compared to haptokinesis. These results are shown in Figure 2A and quantified in Figure 2C. This indicates different adhesive states in the three cell migration modes, leading to changes in cell morphology. Thus, LPA enables a highly adhesive state, while PDGF leads to dimished adhesion to the fibronectin substrate. Evaluation of pseudopods on the bottom filter surface also revealed morphological differences (Figure 2B). In haptotaxis, most protrusions were spread out and covered a large surface. Conversely, a mix of spread and streamlined protrusions (suggesting that they were fixed during a retraction event) were observed in haptokinesis. Consistently with this, a decrease in pseudopod spreading area was observed when haptokinesis was compared to haptotaxis (Figure 2D). During both LPA and PDGF chemotaxis, less spread protrusions were observed (the latter were even less spread than in the haptokinesis experimental paradigm, Figure 2D). Together with the time-lapse experiments mentioned above, these experiments show that the different directional migration systems take advantage of different cell morphologies.

Pseudopod purification was then performed in large scale using the 2-hour postplating time in haptotaxis, and the 1-hour post-stimulation in PDGF and LPA chemotaxis. Representative coomassie stained gels of the pseudopod and cell-body fractions of each cell migration modes are shown in Figure 2E. Finally, we studied spatial activation of MAP kinase ERK signaling in the three cell migration modes (Figure 2F). For that purpose, we used western blot analysis of pseudopod and cell-body fractions equivalents. We observed that both PDGF and LPA gradients lead to sustained ERK phosphorylation, with an increase of phosphoERK signal in pseudopod fractions. This is consistent with the ability of both chemokines to activate ERK [13,14]. As expected, only baseline levels of ERK phosphorylation were detected in haptotaxis. This is consistent with absence of growth factor stimulation. Together, these results indicate the existence of different adhesive states in the three cell migration modes, that correlate with different morphological and signaling states.

### Quantification of pseudopod/cell-body protein enrichment

To get a large scale view of the proteins and the phospho-proteins that are recruited to the pseudopod, we performed quantitative proteomics and phospho-proteomics of the pseudopod and cell-body fractions from the three distinct cell migrations modes. For that purpose, we purified equal amount (1 mg) of pseudopod and cell-body fractions from the three cell migrations modes: haptotaxis, PDGF chemotaxis, LPA chemotaxis, leading to 6 different fractions. Lysates from different experiments were pooled, and were analyzed in three technical replicates, leading to 18 mass-spectrometry runs for each proteomic and phospho-proteomics runs.

Two measures for the quantification of protein abundance in a given fraction were tested: the discrete peptide spectral counts, and the area of the peak associated to a peptide spectrum. Although both measurements were found to correlate (Figure S1A, Additional file 2), the continuous measure of the peptide peak area was used, because of its higher resolution, enabling higher accuracy. Furthermore, the quality of these measurements was evaluated by comparing the standard deviation of the technical triplicates with the mean of the corresponding values (Figure S1B,C, Additional file 2). Different colour coding illustrates that the standard deviation of the measurements ranges in the same levels as the mean values (black) for only 3% of the proteomics, and 2% for the phospho-proteomic measurements, whereas for the majority of the measurements the standard deviation is less than 2-times (red), or less than 10-times (blue) than their corresponding mean value (Figure S1B,C, Additional file 2).

Given that the noise in the measurements scales with the absolute level of detected peptides, the variance of each triplet had to be defined in a scale invariant way. Therefore the coefficient of variation was used as a normalized measure to describe dispersion for the measurement triplicates corresponding to each peptide. Peptide measurements with a coefficient of variation greater than 0.5 were excluded so as to ensure a signal to noise ratio of at least 2 (Figure S1D,E, Additional file 2). Protein information was obtained by taking into account all the peptides or phospho-peptides corresponding to a unique international protein index (IPI) number. To obtain the relative protein or phosphorylation abundance in the pseudopod or cell-body fractions we first grouped all the peptides that correspond to a specific international protein index (IPI), and calculated the median value for each of the technical repeats to avoid the effect of outliers. The ratio of these median values was then calculated for each cell migration mode. For the calculation of these ratios for each experimental condition we further excluded peptides by constraining the sum of coefficients of variation of their technical repeats of each cell migration mode to 0.8, to avoid dividing noisy measurements. For these confident triplets of each cell migration mode the enrichment ratio of their corresponding median was calculated to avoid the bias of any outliers.

Translating the IPI to protein names in both datasets results in 2079 ensemble gene names for the proteomics and 919 for the phospho-proteomic dataset. After applying these filtering conditions we ended up with 1757 unique protein identifiers for the proteomic dataset, and 874 for the phospho-proteomic dataset. Additional information concerning the identification of the proteomic dataset including spectral counts, percentage of protein sequence coverage, and total number of peptides identified for each protein can be found in a supplementary dataset (Additional file 3). Importantly, standardization of our experimental procedure allows for comparison of the relative pseudopod/cell-body enrichment of all these proteins among the three cell migration modes. A list of the proteins and phospho-peptides are provided in the supplementary datasets (Additional files 4 and 5). In our dataset serine phosphorylation is the most prominent phosphorylation event (88.21%), while tyrosine (2.31%) and threonine (9.49%) phosphorylations are much less often observed. Similar proportions were previously reported in Phospho-ELM [15].

**Figure 3:**
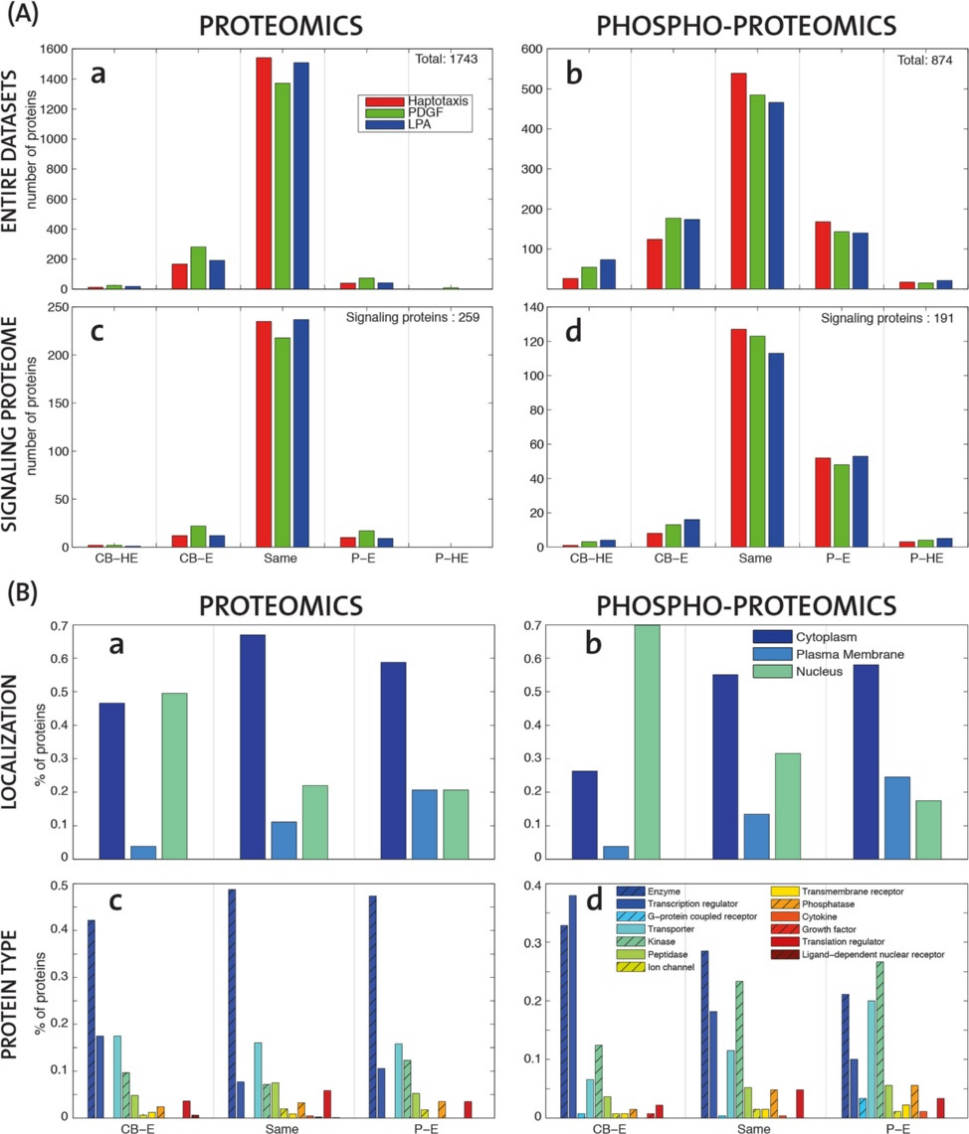
**Subcellular distribution of proteins and phospho-proteins in the different cell migration modes. (A)** Migration-mode dependent distribution of proteins in different cell compartments. Histograms report the number of proteins in the three cell migration modes (Red-HAPTO, Green-PDGF, Blue-LPA). Pseudopod highly-enriched (P-HE) are more than 5-fold enriched in pseudopod; Pseudopod enriched (P-E) are 2 to 5 fold enriched in the pseudopod; Same enrichment (Same) are equally distributed in the cell; Cell-body enriched (CB-E) are 2 to 5 fold enriched in the cell-body; Cell-body highly-enriched (CB-HE) are more than 5-fold enriched in cell-body. The different plots correspond to **(a)** all proteomic dataset (1743 gene names), **(b)** all phosho-proteomic dataset (874 gene names), and **(c)** the subset of proteins (259) or **(d)** phosho-proteins (191) that belong to a previously defined signalling proteome [5]. **(B)** “Spatial” distribution of protein localization and protein types. Percentage of proteins belonging to the three main cellular components **(a,b)**, or the main protein types **(c,d)** with respect to the protein sets belonging to the distinct “spatial” regions of the cell implied by their relative enrichment ranges (as in Figure 3A, without specifying the highly-enriched (HE)) irrespective of cell migration mode.

In order to compare relative enrichment levels, a threshold ratio for enrichment ratio was defined. In Western Blot analysis a 2-fold relative higher intensity is typically taken as indicative of enrichment. Accordingly, in our analysis we considered proteins with enrichment ratios less than 2-fold to be equally distributed between the cell-body-fraction and the pseudopod-fraction, proteins with enrichment ratios of 2 to 5-fold as slightly enriched, and with ratios of more than 5-fold mostly present in one compartment (Figure 3A). As expected, in all three cell migration modes most proteins appear to be equally distributed between the cell-body-fraction and the protrusion-fraction. These most likely represent soluble proteins, that do not have specific localizations in the cell-body or in the pseudopod. Thus, they have homogeneous protein density throughout the cell and will appear as equally distributed when pseudopod/cell-body equivalents are compared. We then observed that there are slightly more proteins that have a biased localization to the cell-body versus the pseudopod (Figure 3Aa). In the phospho-proteome, a large set of phosphorylated proteins were again equally distributed (Figure 3Ab), with slightly more proteins that are present mostly in the cell body.

As a mean of removing proteins that might not be directly related to the signalling responses, apart from the entire dataset (Figure 3Aa,b) we also looked more specifically on the subset of the proteome that has previously been identified as relevant to signaling [16]. In the subset of signaling proteins of the proteomic data, the fraction of equally distributed proteins between compartments is even higher (Figure 3Ac), and there is no clear trend towards enriched or not enriched proteins. However, when we focused on the subset of signalling proteins [17] of the phospho-proteomic dataset a clear enrichment at the protrusion is observed among all stimulation modes (Figure 3Ad). This suggests that the receptors that trigger the different cell migration modes do not alter the overall protein localisation as much as the phosphorylation state of the signaling proteins.

### Quality control of the proteomic and phospho-proteomic dataset using protein classification

The availability of large scale proteomic data allows us to perform a series of internal controls assessing the quality of our dataset using protein metadata from databases. We first analyzed the distribution of the subcellular localization and function of all the proteins and phospho-proteins we identified (regardless of any spatial information deduced from our fractionation scheme), and compared them to a reference dataset consisting of the whole mouse proteome.

Obviously, our proteomic dataset only represents a small subset of the whole mouse proteome. As a quality control, we compared the distribution of subcellular localizations and functions of the proteins and phospho-proteins we identified with those of the whole mouse proteome, ignoring any information about subcellular localization extracted from our fractionation scheme. For that purpose, we simply compared all the proteins and phospho-proteins we identified to a reference protein set that consisted of all the protein names of STRING for *Mus Musculus* (Figure S2, Additional file 2) [17]. The meta-data information regarding subcellular localization and protein type were then extracted using the commercial database software Ingenuity Pathways (IPA - Ingenuity® Systems, http://www.ingenuity.com). Apart from the fact that there were a significant higher number of proteins with unmapped subcellular localization in the whole mouse proteome, we observed a decrease in the number of extracellular proteins in our experimental dataset, which can be because these proteins most likely are washed away during our purification procedure (Figure S2A, Additional file 2). We found an increase of cytoplasmic proteins in the experimental proteomic and phospho-proteomic versus the reference dataset that most likely results from the fact that these proteins are easier to extract and detect using mass-spectrometry. An increase in the occurrence of phosphorylated nuclear proteins was also observed, probably reflecting a large degree of regulation of these proteins by phosphorylation. We then compared the distribution of protein functions present in our experimental versus the reference whole mouse proteome dataset (Figure S2B, Additional file 2). We observed a notable decrease in the amount of GPCRs identified experimentally compared with the whole proteome dataset, which is consistent with the low abundance and low solubility of these membrane proteins [18]. Kinases were observed to be well represented in the experimental proteomic dataset, and were enriched 2-fold in the phospho-proteomic dataset. This implies that kinases are more likely to be found in a phosphorylated state, thus supporting the existence of kinase-based signalling cascades as means of signal transduction. Transcriptional regulators were found in low abundance, but were also enriched in the phospho-proteome, again indicating a large degree of regulation of these proteins. These results indicate that there is no specific bias of our proteomic analysis to proteins with a relatively high representation of some protein types (except for GPCRs). Thus, our experimental dataset faithfully represents a subset of the whole mouse proteome.

### Quality control of biochemical fractionation scheme using protein classification

To control the quality of our cell fractionation scheme, we evaluated the distribution of specific subcellular localization descriptors in the different pseudopod or cell-body fractions. For that purpose, we scored the occurrence of proteins with different localizations (plasma membrane, cytoplasm and nuclear localisation) in the different compartments (Figure 3B a). The proteins sets taken into account here correspond to the union of proteins that follow the same enrichment pattern as in Figure 3A irrespective of cell migration mode. As expected, we observed a bimodal distribution of cytoplasmic proteins, with these proteins being highly abundant in the equally distributed set of proteins, and less abundant in the cell-body and pseudopod fractions. Nuclear proteins were highly enriched in the cell-body and less abundant in the pseudopod. Plasma membrane proteins were enriched in the pseudopod fraction. This might arise because of an increase of plasma membrane in the pseudopod fraction, and/or because of gradient sensing mechanisms that enable directional migration. When the subcellular localization of phospho-proteins was analyzed (Figure 3B b), we observed the same trend for nuclear and plasma membrane proteins. However, in marked contrast with the proteomic dataset, we observed an increase in phosphorylated, cytosolic proteins in the pseudopod. This suggests that phosphorylated cytosolic proteins are recruited to the pseudopod during directed cell migration. Using a similar approach as mentioned above, we then also evaluated the spatial organization of identified proteins and phospho-proteins according to their type (Figure 3B c,d). Because of their low occurrence, highly enriched proteins introduced a statistical bias when subdivided in multiple protein types, and were therefore ignored. An enrichment in kinases at the protein and phospho-protein level was obvious in the pseudopod. This is consistent with a large degree of regulation at this subcellular localization. Importantly, this might also directly results from a bias due to our subcellular fractionation scheme: more efficient identification of kinases might occur because this cell fraction does not contain a lot of the highly abundant proteins involved in the metabolic machinery present in the cell-body that might obscure such low abundant proteins. Transcriptional regulators were abundant in the cell body at the proteomic and phospho-proteomic level, consistently their role at this subcellular localization. Finally, enzymes were not significantly enriched in any fraction at the proteomic level, but showed a clear bias to the cell-body at the phosphoproteomic level. These phosphorylated enzymes most likely represent the metabolic machinery that is regulated within the cell body, and to a lesser extent in pseudopods. These results give further insight into the spatial organization of proteins and phospho-proteins during cell migration.

### Spatial organization of signaling networks regulating cell migration

**Figure 4:**
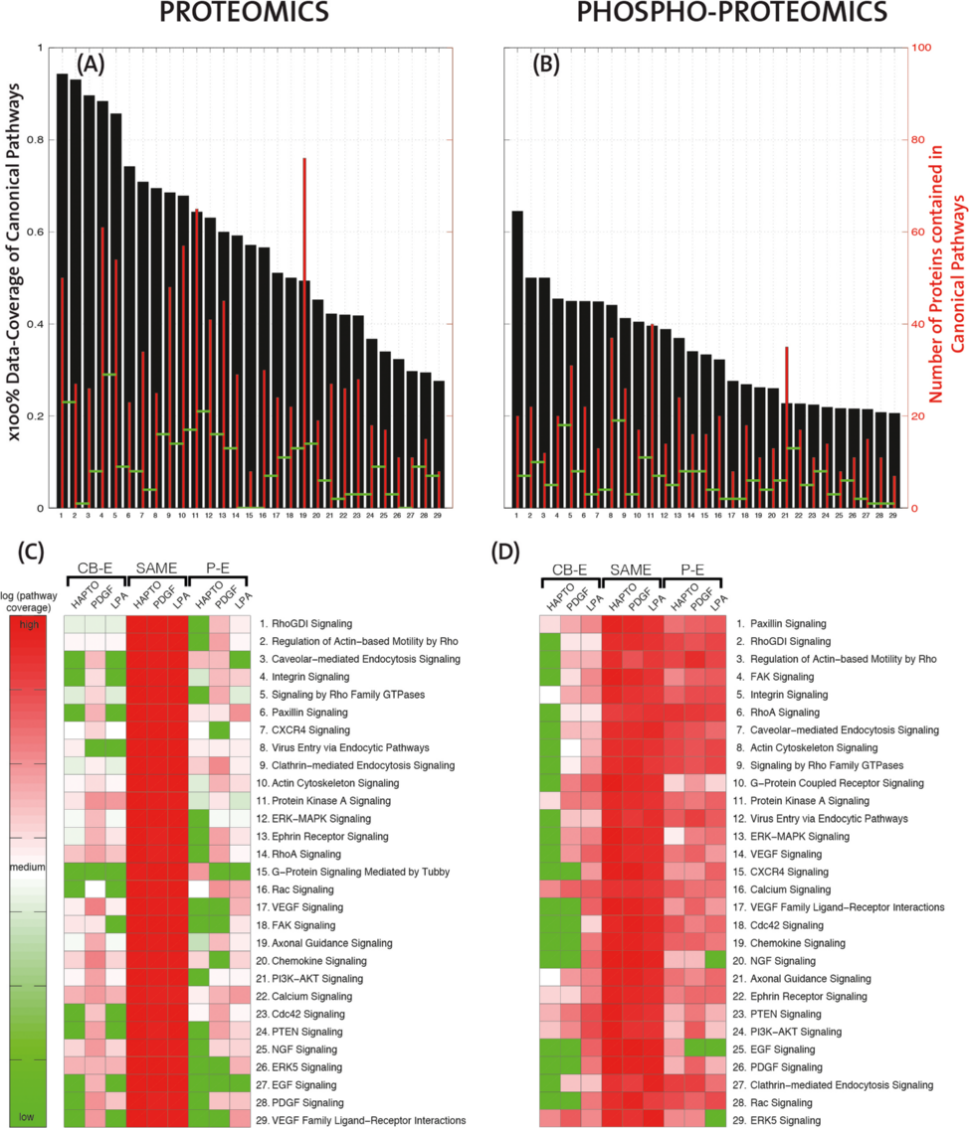
**Spatial organization of cell migration related signalling pathways in the three cell migration modes. (A,B)** Histogram (black) of the coverage (x100%) of cell migration related pathways, ranked in descending order based on the proteomic and phospho-proteomic data sets. For every pathway the absolute number of proteins contained in the data is illustrated with thinner red bars. The level of absolute overlap of subsequent pathways is illustrated with horizontal green segments connecting the red bars. The list of the canonical pathways is indicated on every figure. **(C,D)** Heatmaps of enrichment for the relevant canonical pathways of the proteomic and phospho-proteomic data sets. The pathway names are listed according to descending pathway coverage, and their names correspond exactly to the numbering of panels **A** and **B**. The logarithmic ratio of number of proteins found in each enrichment range, and for every cell migration mode, to the total number of proteins in the data for this pathway, is indicated by a colour map which ranges from green to red. Green corresponds to no proteins and red to all the proteins of this pathway.

We then sought to identify receptor specific spatio-temporal signaling networks inherent to the different cell migration modes. In order to put the proteomic and phospho-proteomic data into a regulatory context, IPA was used to extract information regarding their association with canonical pathways. For that purpose, we selected thirty signaling pathways that are most relevant to signaling and cell migration. We then first evaluated proteomic coverage of these specific signaling pathways (Figure 4A,B, Additional files 6 and 7). Here, the coverage was defined as the fraction of proteins in the data to the total size of each pathway. The coverage ranges from 25% to 95% for the proteomics and from 20% to 65% for the phospho-proteomics. Not all proteins in IPA include canonical pathway annotation, and those that do are usually members of multiple canonical pathways. This data indicates a high degree of coverage of signaling pathways related to signaling and cell migration in our proteomic and phospho-proteomic datasets.

To try to pinpoint specific spatio-temporal signaling networks that are relevant to the different cell migration modes, we evaluated the spatial distribution of these representative "signaling and cell migration" networks in the three cell migration modes. For that purpose, like in Figure 3B, in Figure 4C,D we grouped proteins and phospho-proteins in three categories: Pseudopod-Enriched (P-E) are the proteins that are enriched more than 2 times in the pseudopod, proteins that are distributed the same (SAME) in the two compartments are less than 2 times enriched in the pseudopod or cell-body, and Cell-Body Enriched (CB-E) proteins are enriched more than 2 times in the cell-body. Canonical pathways were ranked according to their signaling pathway coverage. Most proteins of the cell migration pathways were equally distributed (Figure 4C). This stems in part from the higher number of proteins, and thus the higher coverage in this specific fraction. We did not observe clear enrichment of specific signaling networks in pseudopods versus cell-body when all the cell migration modes are considered as a whole. Furthermore we investigated whether proteins follow the same pattern of enrichment among the different modes of migration. Based on the same enrichment thresholds as in Figure 4, we find coherent enrichment among the three modes, as there are only four proteins in the proteomic and one in the phospho-proteomic dataset that are enriched in different fractions for different cell migration modes (Figure S3, Additional file 2); these are all not known to be relevant to cell migration. However, clear cell migration mode specific pathways are found to be enriched in pseudopods. It is important to point out that this results from low data coverage. By example "RhoGDI signaling" indicates no pseudopod-enriched protein in haptotaxis, 4 pseudopod-enriched proteins in PDGF chemotaxis, and 2 pseudopod-enriched proteins in LPA chemotaxis. Furthermore, as the pathways are listed in descending order of their coverage, towards the lower part of the heat map many proteins might be just missing, making the comparison between the three cell migration modes less reliable. Thus, in this proteomic dataset, protein enrichment has to be looked at on a case by case basis, and only gives limited information about true signaling networks. Within our signaling and cell migration protein set, only 10 proteins were found to be pseudopod enriched. In haptotaxis, only IQGAP3, an adaptor protein that regulates Rho GTPase signaling, was found to be pseudopod-enriched specifically in this cell migration mode. Arp2/3 complex subunits (ARPC3, ARPC4, ARPC5L), vascular-endothelial cadherin (CDH5) and the adhesion related adaptor protein CRKL were pseudopod specific in PDGF chemotaxis. The Arp2/3 complex subunit (ARPC4), the CD44 surface glycoprotein, the Ras GTPase K-RAS and the adhesion protein ACTN4 were pseudopod specific in LPA chemotaxis. The functional significance of these proteins will have to be studied on a case by case basis.

When the subcellular distribution of phospho-proteins of this representative "signaling and cell migration" network was evaluated, we found a high level of phosphorylation in equally distributed protein but also pseudopod proteins (Figure 4D). No preferential receptor-specific signaling networks were highlighted, and phospho-proteins therefore have to be evaluated on a case by case basis. Interestingly, there was a lower content of phosphorylated "signaling and cell migration" relevant proteins in the cell body in haptotaxis. This suggests that a sole integrin engagement in haptotaxis leads to lower amount of phosphorylation events in the cell body than when RTK or GPCR signaling crosstalks with integrins during chemotaxis.

### Network context of identified proteins

**Figure 5:**
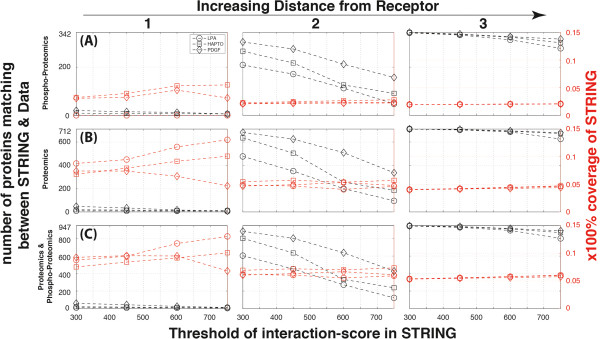
**Network embedding of the three prototypical receptors in the three cell migration modes.** The number (black) and the fraction (red) of proteins for each of the three cell migration modes (squares:HAPTO, diamonds:PDGF, circles:LPA) matching the names of the protein network originating at integrin, RTK and GPCR receptors based on the STRING interaction database for *mus musculus*. The three rows correspond to consideration of different sets of the experimental data: **(A)** Phospho-proteomics, **(B)** Proteomics and **(C)** Proteomics & Phospho-proteomics. Columns correspond to the neighbouring distance of order one, two or three from the receptors of origin.

**Figure 6:**
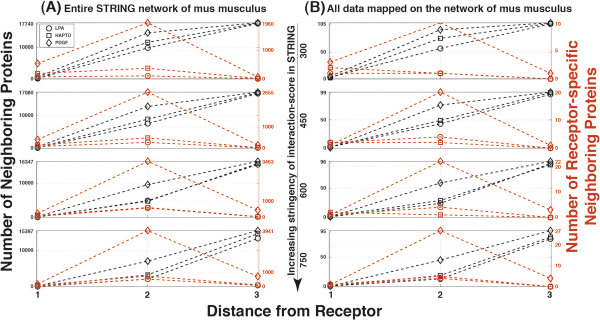
**All neighbouring proteins and receptor-specific proteins based on STRING interaction database. (A)** Number of protein neighbours for distances 1,2 and 3 from each one of the three receptors (squares:ITGA5, diamonds:PDGFRB, circles:LPAR1) according to the entire STRING interaction database for *mus musculus* is indicated either for proteins that are specifically associated with each receptor system (Red), or for proteins that have also shared interactions between the three receptor types (Black). The same figure is illustrated for increasing stringency indicated by the STRING interaction score (300, 450, 600 and 750). **(B)** Same plots for the subset of proteins contained in the entire experimental dataset.

We then took an additional approach to put our specific proteomic datasets in the network context of the different cell migration modes which depend on three prototypical receptors. For that purpose, we evaluated whether proteins known to interact with these receptors, or their downstream effectors, are enriched in pseudopods triggered by receptor-specific cues. Therefore, a graph of interacting proteins was created based on the STRING database. STRING combines information from many different sources on protein-protein interactions [17], and each source provides protein-protein interaction information of different quality, which is reflected in a score that runs between 0 and 999. Using different threshold for the sum (their sum ranges from 300 to 8246) of all these scores we queried the names of all proteins that would interact either with the integrin, the GPCR, or RTK receptors in the STRING database, starting with one representative protein from each prototypical receptor type. We used the fibroblast expressed integrin alpha5 (ITGA5) for haptotaxis, the LPA receptor (LPAR1) for LPA chemotaxis and the PDGF receptor (PDGFRB) for PDGF chemotaxis. We subsequently repeated the query twice to obtain the names of proteins interacting with these binding proteins (distance two from the receptor) and those proteins at distance three from the receptor (Figure 5). STRING reports 17919 protein names in ENSMUSP identifiers for *mus musculus*. By using database dictionaries, the 2357 ensemble gene names in our dataset match 947 ensemble gene names in STRING (342 for the proteomic dataset and 712 for the phospho-proteomic dataset). The coverage of STRING proteins by the data is relatively low. By increasing the threshold of the score, the number of proteins in STRING is reduced, but the coverage remains almost constant, especially for distance two and three from the receptor. Looking only at the proteins that are enriched at the pseudopod more than 2-fold, at least in one mode of migration, the number of proteins is limited even further (Figure S4, Additional file 2). As we can see by using the STRING database, there are more PDGFR related proteins than integrin related ones, and even less LPAR related ones. Given that there is no reference for receptor interconnectivity, this may be just because of the relative amount of studies that has been carried out on each of the systems. The coverage of all the experimental data and every receptor with respect to what is expected from STRING (Figure 5) ranges around 10% for the first order neighbours and drops to 5% for order three as a result of inclusion of irrelevant proteins and high connectivity of the network. To investigate to which extent the different receptor systems can be separated (Figure 6), we excluded all the shared neighbours of the three receptor types, and focused on receptor specific protein sets. Considering either the full STRING database for *mus musculus* (Figure 6A) or the subset corresponding all the experimentally identified proteins (Figure 6B), both for increasing stringencies of the interaction score in STRING, similar trend was observed: the set of specific-receptor associated proteins is low for the first order neighbours, increases for the second order (especially for the PDGF), and drops almost to zero for third order interactions. This shows that the cross-grained scoring of protein interactions in databases does enable yet an analysis at the level of distinct pathway resolution.

### Comparison of relative enrichment of proteins and phospho-proteins

**Figure 7:**
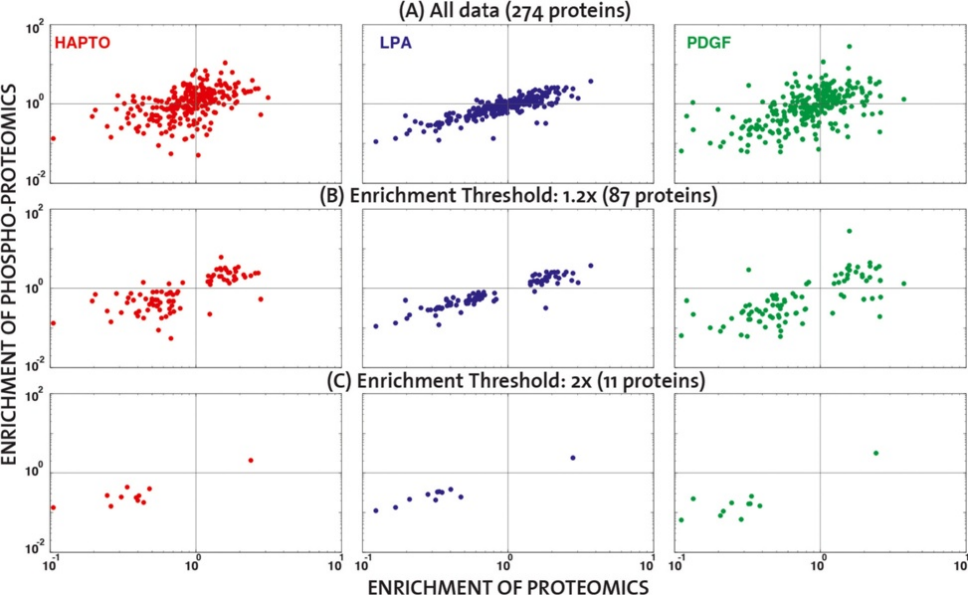
**Correlation of enrichment based on the intersection of proteomic and phospho-proteomic data.** Scatter plots in logarithmic scale of the phospho-proteomic versus the proteomic enrichment for proteins that exist in both datasets. The number of proteins considered varies according to the selected threshold of enrichment: **(A)** All common data (274 proteins), **(B)** 1.2-fold (87 proteins), or **(C)** 2-fold (11 proteins). Colour code: HAPTO red, LPA-blue, PDGF-green.

We then studied if there was any relationship in the subcellular localization of proteins and phospho-proteins. Given that the phospho-proteomics measurement technique has lower sensitivity than the proteomics, only 274 proteins were common to both the proteomic and the phospho-proteomic datasets. Because of the relatively low amount of proteins available for this analysis, we directly correlated enrichment values considering all data) or for protein enrichment values higher than 1.2-fold (Figure 7). We observed an evident correlation between phosphorylation and protein enrichment in all three cell migration modes, implying that proteins that are specifically phosphorylated in the pseudopod or the cell-body are also enriched there. This suggest that most phosphorylated proteins are spatially regulated by immobilization in specific protein complexes serving as subcellular anchors, rather than being locally phosphorylated by reaction diffusion mechanisms involving co-ordinated spatial control of kinases and phosphatases [19].

## Discussion

In this study, we provide for the first time large-scale information about the spatial organization of proteins and phospho-proteins during three different modes of directed cell migration, that involve signaling through distinct prototypical receptors systems: integrins, RTKs and GPCRs. This study makes an important first step towards unravelling the complexity of directed cell migration by underlying the importance of spatially distinct signalling events. For that purpose, we adapted a previously-described pseudopod purification system to three distinct stimuli that engage the different prototypical receptor systems. Our approach has several beneficial features when compared to classic proteomic approaches to study cell signaling in large scale that take advantage of bulk stimulation with ECM components or growth factors. Beyond providing spatial information (relative pseudopod/cell-body enrichment), our approach is very effective at producing robust signaling responses: 1. the response to ECM or chemokine gradients is likely to trigger amplification mechanisms leading to strong signaling responses; 2. the synchronization of membrane protrusion in a specific morphodynamic state (extension) leads to a homogeneous signaling state across the cell population. Thus, it is uniquely suited to study signaling networks downstream of prototypical receptor systems in the context of directed cell migration.

The availability of large scale information about the subcellular localization of proteins and phospho-proteins enables us to understand some emergent properties of spatial regulation of signaling during cell migration. As already previously observed [10,11], we observed an asymmetric subcellular localization of proteins between the pseudopod and the cell-body. As quality control, nuclear and membrane proteins were found to be enriched and phosphorylated in the cell-body and in the pseudopod respectively, indicating that our fractionation scheme allows for enrichment of the right proteins. One interesting insight was that kinases are highly likely to be phosphorylated in the pseudopod, suggesting that during the process of cell migration kinase networks are recruited at that subcellular localization. When correlating the subcellular localization of proteins and their phosphorylated form (n = 274 proteins), we observed that when a protein is enriched at a specific subcellular localization, then it is also phosphorylated there. This suggests that most phosphorylated proteins are spatially regulated by immobilization in specific protein complexes that serve as subcellular anchors.

Because many of the identified proteins are not relevant to cell signaling, we focused our attention on a subset of proteins involved in signaling and cell migration. While we initially anticipated that we would observe robust recruitment of cell migration mode specific signaling networks that might indicate some of their inherent features, we could not identify clear differences between the three cell migration modes. Considering that cell migration is the outcome of an integrated process, the flow of the signal through chemo-receptors needs to be eventually translated into mechanical stress exerted by integrins. Therefore, the overall alignment between the different modes of cell migration is not that surprising. Consistently with the existence of regulation of cytoskeletal and adhesion dynamics in both subcellular compartments, observation of these "signaling and cell migration" related protein ensembles did not show clear segregation to the pseudopod. However, one important limitation is the relatively low coverage that was obtained in our proteomics analysis (1757 proteins total with a large number of proteins being equally distributed between the pseudopod/cell-body fractions). In the future, the increased sensitivity of mass-spectrometry approaches is likely to yield higher protein coverage, in turn allowing a better view of low abundant signaling proteins. In our previous study of the neurite/soma proteomes of neurons with approximately 4800 proteins identified [11], a better coverage in signaling proteins was evident within the neurite, and allowed more efficient identification of canonical pathways.

In contrast to the proteins, the "signaling and cell migration"-related phospho-proteins were clearly biased to the pseudopods. Here again however, we could not distinguish any clear cell migration mode specific patterns. These findings also suggest that signalling stimuli do not alter the overall protein localisation as much as the phosphorylation state of the regulatory proteins, supporting the existence of kinase-based signalling cascades as means of signal transduction.

One important current limitation of our understanding of these spatio-temporal networks still resides in the lack of studies of cell signaling during cell migration with tools that allow understanding the fine regulation of this process. By example, recent evidence has shown that during fibroblast cell migration in response to fibronectin (as in haptotaxis) involve a complex Rho GTPase signaling network [20]. Here, Rac1, Cdc42 and RhoA are activated in highly defined, micrometer-regions that switch ON and OFF on time scales of tens of seconds. In contrast, PDGF-induced fibroblast cell migration (as in PDGF chemotaxis) involves a mode of membrane protrusion without RhoA activity [21]. Consistently, we observe different Rho GTPase regulators that are differentially phosphorylated in the pseudopods depending on the cell migration mode. These most likely regulate these different, dynamic spatio-temporal Rho GTPase signaling networks. Our proteomic dataset might serve as a starting point to understand this complex spatio-temporal modularity. The lack of adequate data on spatio-temporal signaling during cell migration, also explains our inability to identify receptor-specific signaling networks.

In order to place into context all subtle differences between cell migration modes that are encountered throughout the analysis, this study could be followed up by high content imaging experiments that are focused on key proteins. Single node perturbations in a large scale would shed light into causal relationships of protein interactions, and would contribute to a systems understanding of signal integration at the receptor level. This would provide with context specific subgroups of the whole proteomic data. Combining this with manually curated networks would enable the construction of a cell migration interaction map, pointing out key components whose dynamic interactions could then be monitored and studied to further understand their regulatory role.

While still not enabling the understanding of emergent properties of receptor-dependent spatial signaling networks, the general findings of our study provide a comprehensive resource for the cell migration community. Our analysis also gives an overview of the potential, but also the current limitations in the systematic study of proteomic data in a biological context. The results of the analysis for mouse fibroblasts serve as a resource for the general research community, and as a basis for researchers involved with the systemic understanding of biological functions.

## Conclusions

Using a previously established purification-scheme we demonstrated that it is possible to study the spatio-temporal characteristics of signaling downstream of prototypical receptor systems in the context of directed cell migration. By comparing the whole proteomic dataset with its subset, which has previously been characterized as relevant to signalling, we were able to show the bias of phosphorylation events towards the pseudopod of the cell. Moreover, the existence of phosphorylated kinases at the cell front points out the potential compartment-specific role of kinases as means of signal transduction during directed cell migration. Our analysis also illustrates the current limitations in combining such large-scale proteomic datasets with protein interaction databases for further characterization of their role in a network context. The results of this analysis can be used as a resource for further research in fibroblast cell migration assays, and could be taken into account in similar studies of the field.

## Materials and methods

### Cell culture

NIH 3 T3 mouse fibroblasts (American Tissue Culture Collection) were cultured in Dulbecco’s modified Eagle’s medium (DMEM) (Invitrogen) supplemented with 10% fetal bovine serum (FBS), 1% glutamine, and 1% gentamycin. Cells were serum starved in DMEM containing 0.5% FBS overnight before experiments.

### Calibration of membrane protrusion dynamics on transwell filter

To induce directional pseudopod protrusion in response to different extracellular cues, we modified the original assay as shown in Figure 1B [10]. To study the dynamics of membrane protrusion on microporous filters, we used 24 well 3 μm microporous fluoroblock transwell filters (Corning). Filters were first coated with 10 µg/ml fibronectin for one hour at 37°C on one or both sides of the filter. 10^5^ serum-starved NIH 3 T3 fibroblasts previously labeled with Celltracker green (Invitrogen) were plated per well and allowed to attach for one hour. Membrane protrusion from multiple wells was then analyzed simultaneously using live cell imaging on a Nikon Ti Eclipse equipped with a 10× objective, steered by Metamorph software, and using a green fluorescence filter. An autofocus routine that identified the filter pores in brightfield was used to continuously keep focus on the lower filter surface across the different filters. The fluoroblock ensured that only fluorescent signal from the bottom part (from the membrane protrusions) was captured. The experiment was performed for two hours with 5-minute sampling interval. For signal analysis, images were background subtracted; mean fluorescence intensity per field of view was averaged and normalized to fluorescence intensity of the first frame.

For the haptotaxis cell migration model, we only coated the bottom surface of the filter with 10 mg/ml fibronectin, providing the cell that is plated in the top compartment with a "binary" steep gradient of adhesion sites between the top and bottom filter surface. As control, we used a haptokinesis cell migration system, in which both top and bottom filters are coated with 5 mg/ml fibronectin, allowing the cell to freely extend pseudopods on the top and bottom filter surface. For the chemotaxis cell migration system, we coated the top and bottom filter surface with 5 mg/ml fibronectin, and imposed directionality by providing a gradient of PDGF or LPA. This was achieved by adding 20 ng/ml PDGF-BB in serum free medium containing 1% bovine serum albumin (BSA), or 1 μg/ml LPA in serum free medium containing 0.1% BSA in the bottom compartment, allowing the formation of a diffusion gradient from the bottom to the top chamber. As control, a chemokinesis experimental paradigm was created by adding growth factors to both top and bottom compartments.

Because fluoroblock transwell filters are opaque, only the fluorescent signals from pseudopods migrating to the bottom filter surface are observed when imaged with an inverted microscope. All experiments were performed simultaneously allowing for fair comparison between the different cell migration modes. PDGF and LPA were added to the bottom (chemotaxis), or the top and bottom compartments (chemokinesis). No growth factors were added to the hapto -taxis or -kinesis experimental systems. Fluorescence time-lapse imaging was started after one-hour attachment time, when growth factors were added (or not in the case of haptotaxis/haptokinesis experimental paradigms). This enabled the evaluation of membrane protrusion at the bottom filter surface (Figure 1C). An additional movie file shows this in more detail (Additional file 1). Fluorescence intensity per field of view was integrated over time and represents the rate of membrane protrusion on the bottom filter surface (Figure 1D).

### Pseudopod and cell-body purification

For large-scale pseudopod and cell-body purification, 1.5 × 10^6^ serum starved NIH 3 T3 cells were plated on fibronectin-coated transwell filters (2.4-cm wide 3 mm microporous fluoroblock, Corning) in 6 well plates. Cells were allowed to attach for 1 hour, and were then treated (or not) with chemoattractants for an additional hour. The microporous filters were then immediately fixed by a 20-min incubation in ice-cold methanol, and the pseudopods on the filter bottom or the cell bodies on the filter top or where scraped away, using a cotton swab. The remaining structures were then solubilized in the appropriate lysis buffer. For routine Western blot analysis, a 1% SDS buffer containing protease inhibitors and 2 mM Vanadate was used. This typically yielded 30–40 μg of protrusion versus 800 μg of cell-body lysate per 2.4 cm filter, necessitating the pooling of multiple filters for any biochemical experiment. For proteomics studies, cells were lysed in a buffer containing 20 mM Tris pH 7.5, 8 M Urea, 0.1% Rapigest (Waters), 1% vanadate and phosstop phosphatase inhibitor (Roche). 1 mg of each protrusion and cell-body fractions were generated and pooled for the proteomics analysis.

### Sample preparation

The samples were vortexed, sonicated at 4°C (Hielscher), shaked for 5 minutes on a thermomixer (Eppendorf) and centrifuged for 20 minutes at 4°C and 16,000 g. Supernatants were collected and stored at -80°C for further processing. BCA Protein Assay (Pierce) was used to measure protein concentration. Disulfide bonds were reduced with tris(2-carboxyethyl)phosphine (Sigma) at a final concentration of 10 mM at 37°C for 1 hour. Free thiols were alkylated with 20 mM iodoacetamide (Sigma) at room temperature for 30 minutes in the dark. The excess of iodoacetamide was quenched with N-acetyl cysteine (Sigma) at a final concentration of 25 mM for 10 minutes at room temperature. The solution was subsequently diluted with 0.1 M ammoniumbicarbonate (Sigma) to a final concentration below 2 M urea and digested overnight at 37°C with sequencing-grade modified trypsin (Promega) at a protein-to-enzyme ratio of 50:1. Peptides were desalted on a C18 Sep-Pak cartridge (Waters) and dried under vacuum. A small aliquot of the peptides (10%) was taken for protein abundance measurements while the remaining 90% were subjected to phosphopeptide analysis using TiO_2_ as described previously [22]. Briefly, dried peptides were dissolved in an 80% acetonitrile (ACN)–2.5% trifluoroacetic acid (TFA) solution saturated with phthalic acid. Peptides were added to the same amount of equilibrated TiO_2_ (5-μm bead size, GL Science) in a blocked Mobicol spin column (MoBiTec) that was incubated for 30 minutes with end-over-end rotation. The column was washed twice with the saturated phthalic acid solution, twice with 80% ACN and 0.1% TFA, and finally twice with 0.1% TFA. The peptides were eluted with a 0.3 M NH_4_OH solution. The pH of the eluates was adjusted to be below 2.5 with 5% TFA solution and 2 M HCl. Phosphopeptides were again desalted with microspin C18 cartridges (Harvard Apparatus).

### LC-MS/MS analysis

The setup of the μRPLC-MS system was as described previously [23]. The hybrid LTQ-Orbitrap XL mass spectrometer was interfaced to a nanoelectrospray ion source (both Thermo Electron, Bremen, Germany) coupled online to a Tempo 1D-plus nanoLC (Applied Biosystems/MDS Sciex, Foster City, CA). 1 μg of total peptide mass was separated on a RP-LC column (75 μm × 15 cm) packed in-house with C18 resin (Magic C18 AQ 3 μm; Michrom BioResources, Auburn, CA, USA) using a linear gradient from 96% solvent A (98% water, 2% acetonitrile, 0.15% formic acid) and 4% solvent B (98% acetonitrile, 2% water, 0.15% formic acid) to 30% solvent B over 120 minutes at a flow rate of 0.3 μl/min. Each survey scan acquired at 60,000 FWHM was followed by MS/MS scans of the five most intense precursor ions in the linear ion trap with dynamic exclusion enabled for a period of 60 seconds. Charge state screening was employed to select for ions with at least two charges and rejecting ions with undetermined charge state. The normalized collision energy was set to 32% and one microscan was acquired for each spectrum.

### Database searching

After converting the acquired raw files to the centroid mzXML format using ReAdW (*http://tools.proteomecenter.org/wiki/index.php?title=Software:ReAdW*), MS/MS spectra were searched using the SORCERER-SEQUEST™ v4.0.3 algorithm against a decoy database (consisting of forward and reverse protein sequences) of the predicted proteome MOUSE (IPI, *http://www.ebi.ac.uk*, release date: 16/05/2011) and commonly observed contaminants. The search criteria were set as follows: semi-tryptic specificity was required (cleavage after lysine or arginine residues, unless followed by proline, one tryptic termini required); 2 missed cleavages were allowed; carbamidomethylation (C) was set as fixed modification; oxidation (M) was applied as variable modification; in case of phosphopeptide enriched samples, phosphorylation (S,T,Y) was added as a variable modification; mass tolerance of 15 ppm (precursor) and 0.8 Da (fragments). The database search results were further processed using the PeptideProphet [24] and ProteinProphet [25] program and the peptide false discovery rate (FDR) was set to 1% on the peptide and protein level and validated using the number of reverse protein sequence hits in the datasets.

### Label-free quantification

The acquired raw-files were imported into the Progenesis software tool (Nonlinear Dynamics, Version 3.0) for label-free quantification using the default parameters. After feature extraction and alignment, the database search results (in pepxml format) were imported to the Progenesis software for MS1 feature assignment. A csv-file containing the MS1 peak abundances of all detected features was exported and further processed using in-house software. In brief, the software sets the peptide/phosphopeptide/protein identification level False Discovery Rate to 1% (based on the number of decoy protein sequence database hits) and normalizes the identified MS1 peak abundances across all samples.

The method is label free but provides precise quantitation of the abundance of different proteins in the complex mixture. For each fraction, a list of proteins using the international protein index (IPI) identification system is provided with information about its abundance. Because the mass of the pseudopodia is only a small fraction of that of the whole cell, our purification scheme does not permit the analysis of pseudopodia/cell-body equivalents. Hence, assuming that the global protein density is equal in both sub cellular domains, equivalent amounts of pseudopodia and cell-body lysate are compared. The analysis thus yields information regarding the relative abundance of proteins. Proteins that are either enriched or depleted in the pseudopodia will be identified through comparison of protein densities in pseudopodia and cell-body. One important assumption we make is that protein density is constant throughout the cell.

### Proteomic and phosphoproteomic datasets

The proteomic analysis has been performed using a novel mass spectrometry method that enables the rapid characterization of complex peptide mixtures without bias to highly abundant proteins [26]. For the proteomic analysis equal amount (10 μg) of pseudopod and cell-body fraction were analyzed by mass spectrometer. The proteomic dataset consists of 2103 peptides, that are all identified in the 18 different mass-spectrometry runs, with a total sum of 12759 peptide counts. For the phosphoproteomic analysis, 1 mg of pseudopod and body lysates were digested and subjected to phosphopeptide analysis using TiO_2_ affinity purification as previously described [22], resulting in 2074 phosphorylated peptides. Provided that the proteomic and phosphoproteomic analyses are independent, and have different sensitivities, their identification output is not necessarily the same.

## Abbreviations

ECM: Extracellular matrix; RTK: Receptor tyrosine kinase; GPCR: G-coupled protein receptors; PDGF: Platelet derived growth factor; LPA: Lysophosphatidic acid.

## Competing interests

The authors declare that they have no competing interests.

## Authors’ contributions

OP, KM and EF performed the biological and biochemical experiments. RA provided the infrastructure for mass spectrometry and AS performed the high-throughput experimental analysis of the cell lysates using mass spectrometry. GF did the data analysis with DI and OP as advisors. GF, DI and OP wrote the manuscript. All authors read and approved the final manuscript.

## Supplementary Material

Additional file 1 **Timelapse of pseudopodial extension through the filter pores for PDGF and LPA-mediated NIH 3 T3 chemotaxis and fibronectin-mediated haptotaxis.** Bar = 50 μm. Click here for file

Additional file 2 Supplementary figures and legends. Click here for file

Additional file 3 **Identification information of Proteomic dataset.** The ensemble gene names of the identified proteins are listed in column 1, and the spectral counts in column 2, a measure that determines the relative protein amounts. Column 4 shows the percentage of protein sequence coverage, indicating the percentage of the protein's sequence represented by the peptides identified in the mass spectrometry run, and in column 5 is the total number of peptides identified for each protein. Click here for file

Additional file 4 **Proteomic dataset.** The ensemble gene names of the identified proteins are listed in column 1, and their relative enrichment ratios in each cell migration mode (Haptotaxis, LPA, PDGF) in columns 2–4. In the following columns a binary matrix indicates protein localization (columns 5–10), protein type (columns 11–24), and signalling pathways (columns 25:225). The last three columns (226–228) contain the p-values as determined by a ttest comparing the mean enrichment values of the cell protrusion versus the mean enrichment values of the cell body, for each cell migration mode (Haptotaxis, LPA, PDGF), under the null-hypothesis that they are equal. Click here for file

Additional file 5 **Phosphoproteomic dataset.** The ensemble gene names of the identified phosphoproteins are listed in column 1, and their relative enrichment ratios in each cell migration mode (Haptotaxis, LPA, PDGF) in columns 2–4. In the following columns a binary matrix indicates protein localization (columns 5–10), protein type (columns 11–25), and signalling pathways (columns 26:235). In column 236, the phosphorylated peptide(s) with phosphorylation site annotation is provided. The index of every phosphorylated aminoacid (Serine:S,Threonine:T,Tyrosine:Y) is indicated in brackets for each peptide, together with the detected change of its mass. The last three columns (237–229) contain the p-values as determined by a ttest comparing the mean enrichment values of the cell protrusion versus the mean enrichment values of the cell body, for each cell migration mode (Haptotaxis, LPA, PDGF), under the null-hypothesis that they are equal. Click here for file

Additional file 6 **List of identified proteins and their correspondence to selected canonical pathways related to cell migration.** A binary matrix indicates the ensemble gene names of the proteomic dataset belonging to the manually selected cell migration related canonical pathways according to IPA. Click here for file

Additional file 7 **List of identified phosphoproteins and their correspondence to selected canonical pathways related to cell migration.** A binary matrix indicates the ensemble gene names of the phosphoproteomic dataset belonging to the manually selected cell migration related canonical pathways according to IPA. Click here for file
