# Supplementary material for: Spatial proteomic and phospho-proteomic organization in three prototypical cell migration modes
Source: Proteome Sci. 2014 May 1;12:23. doi: 10.1186/1477-5956-12-23 (PMC4077045; doi:10.1186/1477-5956-12-23)
Supplement: Additional file 2 — Supplementary figures and legends. [file 1477-5956-12-23-S2.pdf]

Supplementary Figures and Legends

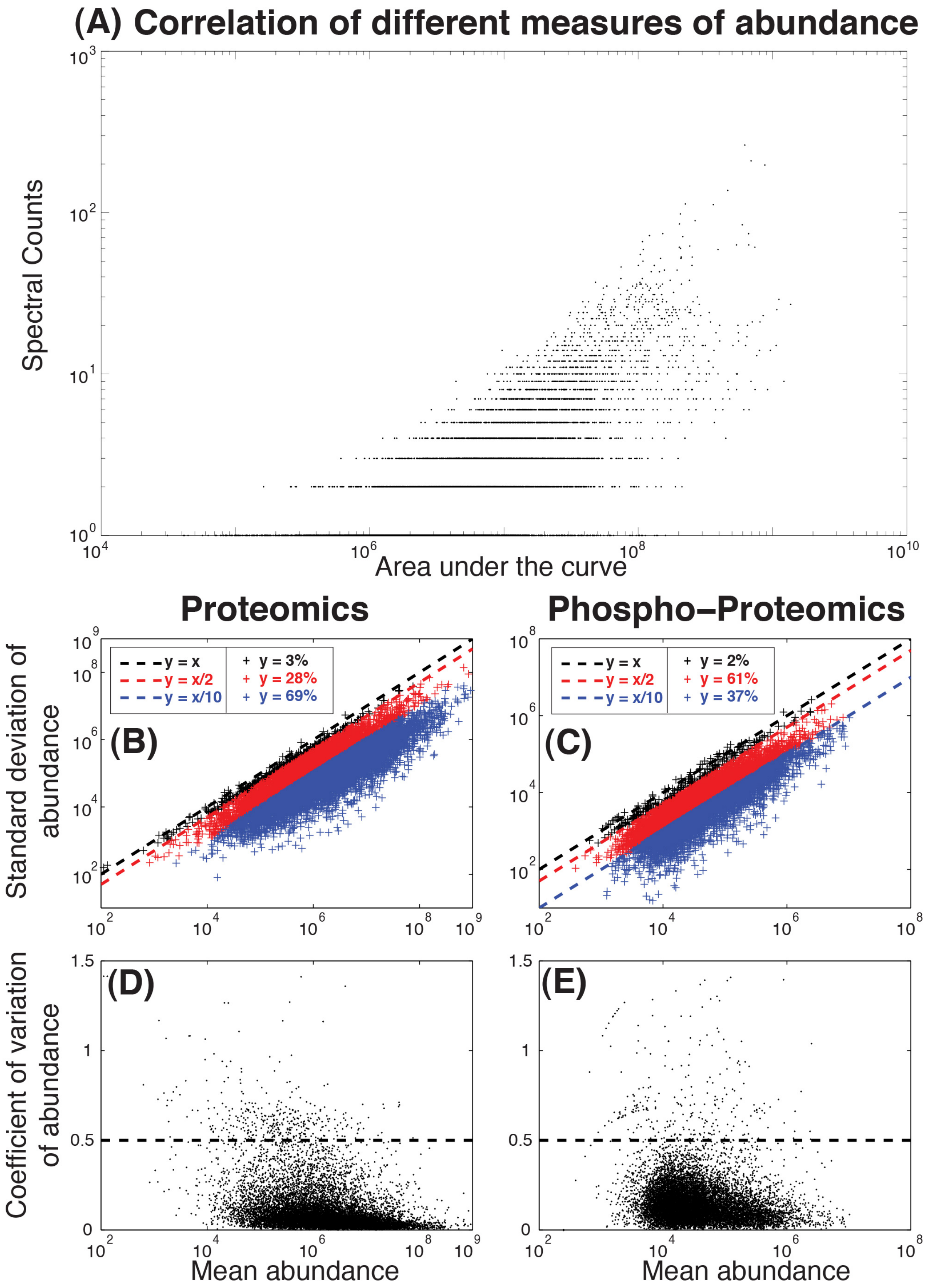

Figure S1. Quality assessment of proteomic and phospho-proteomic data. (A) Correlation of the two measures quantifying protein abundance: number of spectral counts and area associated to the spectra of peptides. **(B)** For every triplicate of our measurements, a cross

point illustrates the standard deviation of the abundance (y-axis) versus the mean value of the abundances (x-axis) for the proteomic and **(C)** the phospho-proteomic data. Three dashed lines of different colour illustrate regions for the relation of the standard deviation (y-axis) with the corresponding mean values (x-axis). Cross points lying between the black and red lines have standard deviation which ranges from equal to half of their mean (for 3% of the proteomics, and 2 % of the phospho-proteomics), cross points lying between the red and blue lines have standard deviation which ranges from half to a tenth of their mean (28% and 61% respectively), and cross points below the blue line have standard deviation which is less than a tenth of their mean (69% and 37% respectively). **(D)** Coefficient of variation for the technical triplicates of the proteomic and **(E)** the phospho-proteomic data.

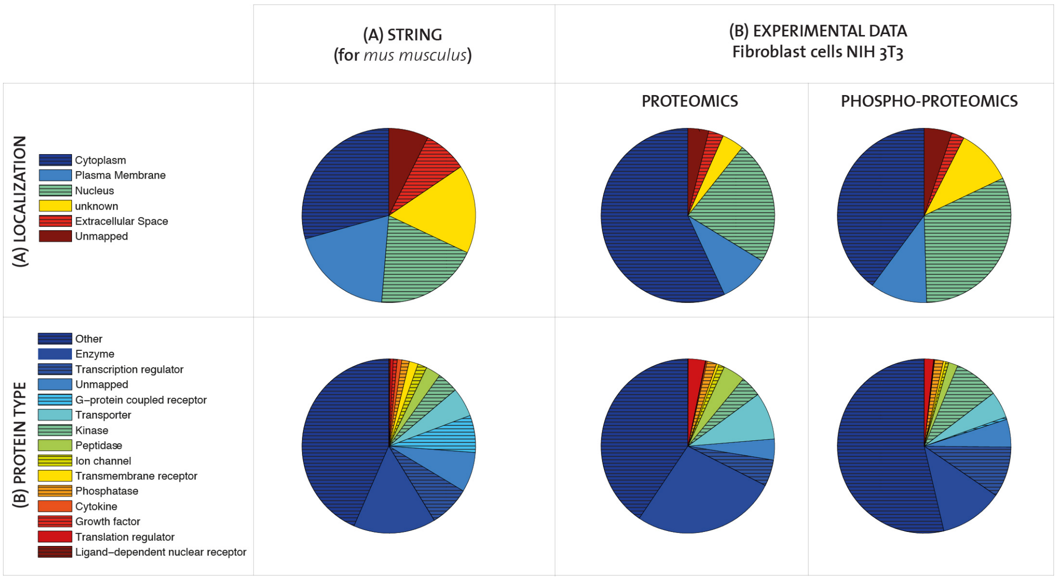

Figure S2. Characterization of protein subcellular distribution and function of proteomic and phospho-proteomic datasets compared to a reference "whole mouse proteome" dataset. Pie charts for the proportions of proteins with specific subcellular distributions (**A**) and protein types **(B)** according to IPA are shown. An ensemble of all the protein names of STRING for *mus musculus* (reference "whole mouse proteome" dataset), and for the proteomic and the phospho-proteomic experimental data sets. Proteins that are not assigned to a particular cellular component are marked as “unknown”, and those that do not belong to one of the main protein types of IPA as “other”. Proteins that were not registered in IPA are marked as “unmapped”.

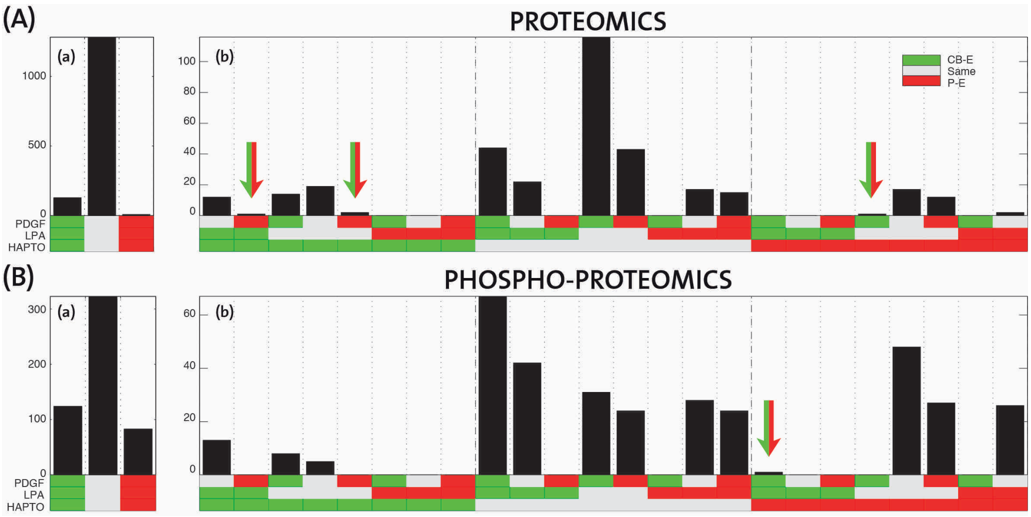

Figure S3. Homogeneous and heterogeneous regulation patterns among the three different cell migration modes. Histograms for the number of proteins corresponding to each pattern of enrichment for each of the three different cell-migration modes. The horizontal coloured bar at the basis of the histogram indicates the distinct enrichment patterns, with each row from bottom to top corresponding to HAPTO, PDGF and LPA migration modes. Both the sets of proteomics **(A)** and phospho-proteomics **(B)** are separated in homogeneous **(a)** and heterogeneous **(b)** patterns of enrichment. Colormap of each mode: green-CB-E: more than 2-fold enriched at the cell-body-fraction, white-Same: equally distributed, red-P-E: more than 2-fold enriched at the protrusion. Double-coloured arrows (green and red) indicate low populated patterns, which include simultaneous enrichment of protrusion and cell body in different cell migration modes.

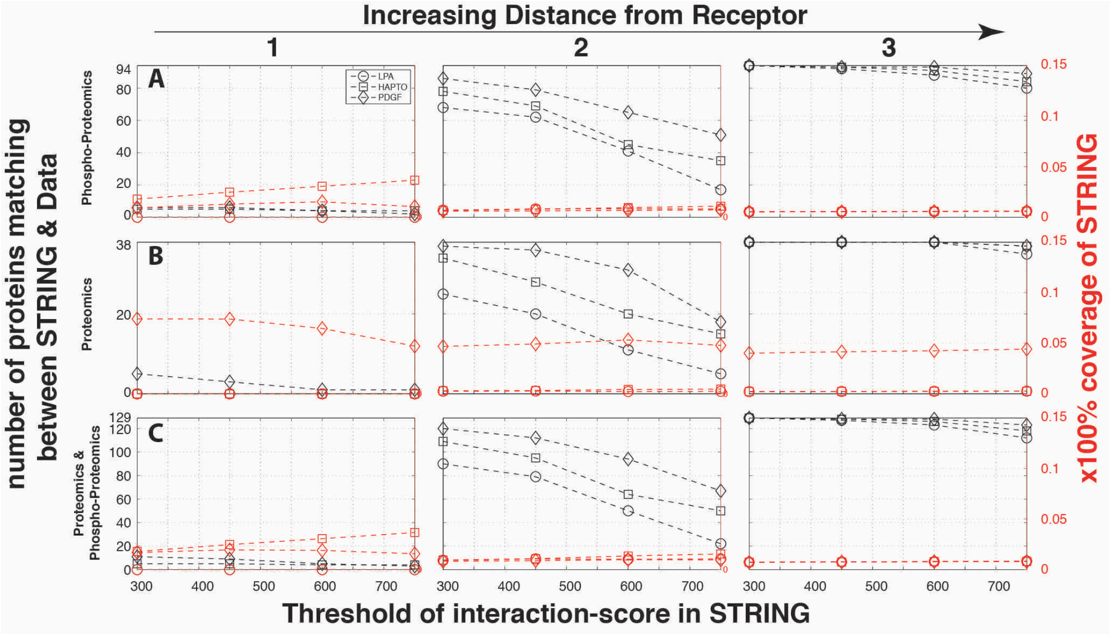

Figure S4. Network embedding of the three distinct receptor systems for the pseudopod-enriched proteins. Similar to Figure 4, for the subset of proteins enriched at the pseudopod more than 2-fold at least in one mode of migration.

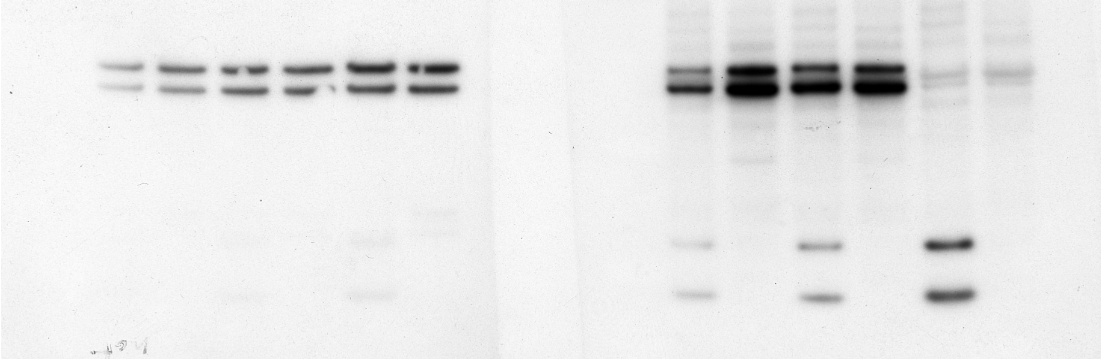

Figure S5. Non-cropped, raw western blot data from Figure 2F. Left panel: total-ERK. Right panel: phospho-ERK.
